# High-Intensity Training on Patients with Hypertrophic Cardiomyopathy: A Systematic Review

**DOI:** 10.1186/s43044-025-00642-2

**Published:** 2025-05-15

**Authors:** Catalya Christina Cantika, Muhammad Nur Hidayah Rayhan Wiradiharja, Lufthi Fahreza, Cici Irawanti Putri, Verousson Ahmad, Muhana Fawwazy Ilyas, Matthew Aldo Wijayanto, Adigama Priamas Febrianto

**Affiliations:** 1 Krida Wacana Christian University, Jakarta, Indonesia; 2 Sekarwangi Regional Hospital, Sukabumi, Indonesia; 3 Alimuddin Umar Regional Hospital, West Lampung, Indonesia; 4 Ujung Gading General Hospital, West Pasaman, Indonesia; 5 University of Brawijaya, Malang, Indonesia; 6 Universitas Sebelas Maret, Surakarta, Indonesia; 7 Department of Cardiology and Vascular Medicine, Sleman General Hospital, Yogyakarta, Indonesia

**Keywords:** Cardiomyopathy, Exercise, High-intensity exercise, Hypertrophic cardiomyopathy, Systematic review

## Abstract

Hypertrophic cardiomyopathy (HCM) is a common inherited cardiac disorder associated with significant morbidity. The role of high-intensity exercise in HCM remains controversial due to concerns about potential adverse effects. This study aims to conduct a systematic review of experimental and observational studies to evaluate the effects of high-intensity training (HIT) on patients diagnosed with HCM. This systematic review was conducted according to the Preferred Reporting Items in the Systematic Review and Meta-analysis Guidelines 2020. A comprehensive search was performed using Proquest, Pubmed, Scopus, and JSTOR databases to identify relevant studies published up to March 2024. Both experimental and observational studies written in English were included in the study. No restrictions were placed on the year, country of publication, or duration of follow-up. Study quality was evaluated using the Effective Public Health Practice Project (EPHPP) tool. A narrative synthesis was also conducted to summarize the findings. A total of 1418 references were identified through the search strategy. After removing 151 duplicates, 1267 records were screened based on title and abstract, resulting in the exclusion of 1253 studies. The full text of 14 articles was assessed for eligibility, of which ten were excluded. Four papers with a total of 2008 sample sizes were included. Significant differences were observed in several echocardiographic parameters. HCM athletes exhibited higher left ventricular end-diastolic volume (96 ± 33 vs. 76 ± 27 mL; *p* = 0.001, effect size = 0.682) and stroke volume (58 ± 21 vs. 47 ± 17 mL; *p* = 0.004, effect size = 0.593) compared to non-athletes. Additionally, significant differences were found in left ventricular outflow tract (LVOT) gradients: both resting (*p* < 0.001, effect size = − 0.192) and provoked (*p* < 0.001, effect size = − 0.258) LVOT gradients were significantly lower in the vigorous exercise group compared to the non-vigorous exercise group. In contrast, no significant differences were found between HIT and the comparator group in vital signs and functional aerobic capacity. Moreover, no significant differences were observed in arrhythmias, cardiac arrest, or mortality, suggesting that HIT did not increase the risk of arrhythmias or other adverse events compared to the comparator group. HIT improves cardiac function and exercise capacity in patients with HCM without increasing the risk of severe adverse events. These findings support reconsidering the guidelines on physical activity restrictions for individuals with HCM.

## Background

Hypertrophic cardiomyopathy (HCM) often leads to impaired cardiac function and heightened susceptibility to life-threatening complications such as heart failure and sudden cardiac death [1, 2]. This condition represents a significant challenge in clinical management, necessitating a further understanding of its pathophysiology and optimal therapeutic strategies. One area of particular contention in managing HCM is the role of high-intensity exercise in individuals with this condition. Patients with HCM have traditionally been prohibited from engaging in vigorous physical activity, including competitive sports and high-intensity exercise [3]. This is possibly due to concerns regarding the potential exacerbation of cardiac symptoms and increased risk of adverse cardiovascular events.

However, recent research has shed light on the complex interplay between exercise and cardiac health in individuals with HCM, prompting a reevaluation of existing guidelines and clinical practices. Previous meta-analysis studies showed that high-intensity interval training (HIIT) improves cardiorespiratory capacity in individuals with coronary artery disease, heart failure, hypertension, metabolic syndrome, and obesity by nearly twice as much as maximal cardiopulmonary training [4]. In terms HCM, guidelines have been revised in the past decade due to improved quality evidence on the benefits and risks of exercise training. Recreational exercise with a mild to moderate intensity now has a Class I indication, while competitive sports and high-intensity exercise have a Class 2b [5].

Another study showed that athletes with genetic heart problems could maintain their competitiveness with a relatively low frequency of cardiac events provided a thorough treatment plan is implemented, and a collaborative decision on potential risks is made [6]. Several studies on exercise training in HCM have been conducted previously; however, the conclusion regarding the safety and efficacy of high-intensity exercise in this population remains unclear. A randomized controlled trial showed that both high-intensity training (HIT) and moderate intensity training (MIT) increased fitness in HCM, although there were no differences between both groups [7]. In addition, another trial, which offers the largest assessment of exercise safety in HCM to date, recently showed no difference in rates of sudden cardiac arrest among individuals with HCM who report strenuous activity than those exercising moderately or sedentary [8]. In another cohort study of low-risk HCM athletes, choosing to continue participating in competitive sports voluntarily was not linked to a higher risk of major cardiac events or deterioration of clinical condition compared to reducing or stopping participating in sports and exercise programs [9].

Although these findings are promising, most studies have been underpowered to demonstrate the safety of HIT in HCM patients due to the rarity of the adverse event [7]. This study aims to conduct a systematic review of experimental and observational studies to evaluate the effects of high-intensity exercise on patients diagnosed with HCM. Therefore, utilizing this approach has the potential to inform clinical decision-making, enhance patient counseling on high-intensity exercise safety, and ultimately contribute to improved health outcomes for patients with HCM.

## Methods

### Study design

This study was conducted in accordance with the Preferred Reporting Items for Systematic Reviews and Meta-Analyses (PRISMA) 2020. The review protocol was registered on the International Prospective Register of Systematic Reviews (PROSPERO) with ID: CRD42024561359. The search was carried out in March 2024 using four international databases: Proquest, PubMed, Scopus, and JSTOR. Search terms for relevant scientific studies were derived from medical subject headings (MeSH).

### Eligibility criteria

We included studies that fulfilled the following eligibility requirements: (1) The studies must be peer-reviewed original articles in the English language; (2) the study design must be experimental (randomized controlled trial (RCT) or quasi-experimental studies) or observational studies (cohort, case–control, or cross-sectional); (3) participant are patient with hypertrophic cardiomyopathy according to Americal Heart Association (AHA) criteria [5], which received HIIT or its equivalent, including strenuous exercise or athletes training; (4) there is at least one of outcome studied, including cardiac function parameters (left ventricular ejection fraction (LVEF), left ventricular end-diastolic volume (LVEDV), left ventricular diastolic diameter (LVDD), stroke volume (SV), or global longitudinal strain), performance of the cardiopulmonary organs (maximum oxygen uptake (VO2 max), heart rate (HR), systolic blood pressure (SBP), diastolic blood pressure (DBP), electrocardiography changes, or safety profiles (mortality or cardiac arrest)). We did not limit the date, geographic location of the studies, and certain characteristics of the participants, such as gender, age, and race.

### Study selection

The results of the preliminary study search were screened. After removing duplicates by looking at the title and abstract, we classify these manuscripts as included or not included. Subsequently, the full text of each article included was assessed. Four reviewers assessed the entire study selection processes blindly and independently (CCC, LF, CIP, and VA). Any discrepancies across all reviews are resolved by discussion with the fifth reviewer (MNHRW).

### Quality assessment

The Effective Public Health Practice Project (EPHPP) was utilized to evaluate the quality of studies included [10]. Six criteria are used: selection bias, study design, confounders, blinding, data collection techniques, and withdrawals or dropouts. There are two questions for each criterion—a total of 14—apart from the"study design"component, which contains four questions (if RCT). The rate (weak, moderate, and strong) represents the outcome of each criterion. The study's global rating (weak, moderate, and strong) is then determined by considering the rates of all six criteria. Four reviewers (CCC, LF, CIP, and VA) blindly and independently assess the quality of studies. Any discrepancies across all reviews are resolved by discussion with the fifth reviewer (MNHRW).

### Data extraction and analysis

The data analysis in this systematic review consisted of a qualitative approach. The data extracted were the author, year of publication, country, study design, sample size, intervention, comparison, and outcomes. Dichotomous data (frequencies and percentages) and continuous/numerical data (Mean ± SD) of each outcome were retrieved from included studies. Additionally, *p*-values for all variables were directly retrieved from the original study data. Effect sizes for continuous data were computed using Cohen’s h. For binary data, effect sizes were computed using odds ratio. Subsequently, a qualitative approach was performed by summarizing the findings of the included studies using a narrative synthesis technique. Four reviewers performed the entire process blindly and independently (CCC, LF, CIP, and VA). Any discrepancies across all reviews are resolved by discussion with the fifth reviewer (RW).

## Results

### Study characteristics

**Fig. 1:**
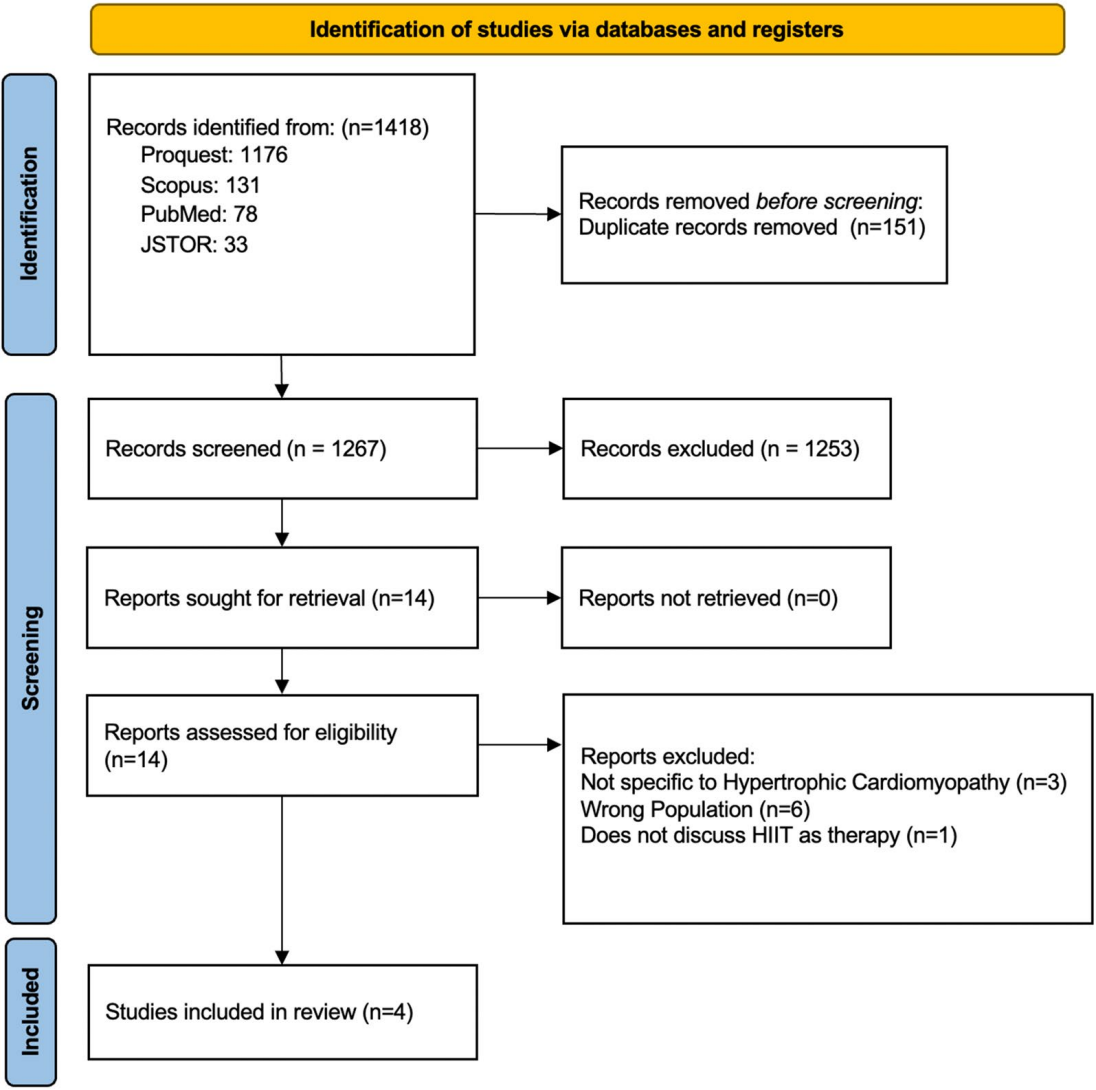
Identification of studies via databases and register

The study selection process of this review followed the PRISMA guidelines. Four databases (Proquest = 1176; Scopus = 131; PubMed = 78; JSTOR = 33) collected 1418 studies retrieved from these databases. Subsequently, 151 duplicated records were removed before the screening process. Based on the screening of titles and abstracts, we excluded 1253 records. We assessed the eligibility of the remaining 14 reports in full text. We excluded six studies due to irrelevant populations, three studies not specific to HCM, and one study that does not discuss HIIT as therapy. Last, the final selection included four articles in this review, as shown in Fig. 1. The study characteristics can be shown in Table 1. Subsequently, quality assessment results are described in Table 2.

**Table 1:** Descriptive characteristics of included studies

No.	Authors	Country	Study design	Total subjects	Intervention and comparison
1	Dejgaard et al. [11]	Norway	Cross sectional	187	I: Athlete C: Non-athlete
2	Pellicia et al. [9]	Italy	Retrospective	88	I: Continued exercise/sport C: Reduced or stopped exercise/sport
3	MacNamara et al. [7]	United States	RCT	15	I: High-intensity training (HIT) C: Moderate intensity training (MIT)
4	Lampert et al. [8]	United States	Cohort	1660	I: Vigorous exercise C:Nonvigorous activity

**Table 2:** Quality assessment of included studies

No.	Authors	Selection Bias	Study design	Confounders	Blinding	Data collection method	Withdrawal and dropouts	Overall rating
1	Dejgaard et al. (2018) [11]	Moderate	Weak	Weak	Weak	Moderate	Not Applicable	Weak
2	Pellicia et al. (2019) [9]	Moderate	Moderate	Weak	Weak	Moderate	Not Applicable	Weak
3	MacNamara et al. (2023) [7]	Moderate	Strong	Weak	Moderate	Strong	Moderate	Moderate
4	Lampert et al. (2023) [8]	Moderate	Moderate	Weak	Moderate	Moderate	Weak	Weak

### Definition of high-intensity training

HIT is broadly defined as vigorous exercise that requires a high level of exertion, typically measured by metabolic equivalents (METs) or heart rate thresholds. Dejgaard et al. [11] defined vigorous exercise as ≥ 6 METs, equivalent to jogging, rated using the Compendium of Physical Activities. Athletes were then defined as those with ≥ 4 h/week of vigorous exercise for ≥ 6 years, and competitive athletes were those participating in organized sports or competitions [11]. Lampert et al. [8] similarly defined HIT as activity ≥ 6 METs, performed consistently over time. MacNamara et al. [7] specified HIT as structured interval training at 90–95% of peak heart rate, involving repeated 4-min high-intensity bouts separated by 3-min recovery periods, conducted over 4 months following a month of moderate intensity preparation. Last, Pelliccia et al. [9] described HIT as regular athletic training lasting two or more hours per session, three times a week, with total weekly vigorous exercise ranging from 6 to 14 h, often involving participation in competitive sports.

The comparator groups across these studies consistently engaged in lower intensity exercise, typically classified as moderate intensity or below. Dejgaard et al. [11] and Lampert et al. [8] described the comparator groups as non-athletes or individuals engaging only in leisure activities below 6 METs. MacNamara et al. [7] contrasted HIT with MIT, defined as continuous aerobic exercise below the maximal steady state without interval training. Pelliccia et al. [9] classified the comparator group as"detrained"individuals with HCM who had either ceased or significantly reduced vigorous exercise, engaging in less than 6 h of exercise per week. Across all studies, HIT involved higher exertion levels and structured protocols, while comparator groups were defined by lower intensity, non-competitive, or recreational physical activity.

### Effect and safety profile of high-intensity training

No significant differences were found between HIT and the comparator group in terms of vital signs and functional aerobic capacity. Specifically, SBP, DBP, HR max, and VO2 max did not show statistically significant changes (all *p* > 0.05), indicating that HIT did not significantly impact these outcomes compared to the comparator group. However, significant differences were observed in several echocardiographic parameters. HCM athletes had higher LVEDV (96 ± 33 vs. 76 ± 27 mL; *p* = 0.001, effect size = 0.682) and SV (58 ± 21 vs. 47 ± 17 mL; *p* = 0.004, effect size = 0.593) compared to non-athletes. Additionally, both resting (18.3 ± 22.3 vs. 23.0 ± 25.9; *p* < 0.001, effect size = − 0.192) and provoked (39.6 ± 42.0 vs. 51.1 ± 46.3; *p* < 0.001, effect size = − 0.258) LVOT gradients were significantly lower in the vigorous exercise group compared to the non-vigorous exercise group (Table 3). Last, no significant differences in arrhythmias, cardiac arrest, or mortality were found between the HIT and comparator groups (Table 4), with no difference in the incidence of non-sustained ventricular tachycardia (NSVT), sustained ventricular tachycardia, ventricular fibrillation, or syncope (all *p* > 0.05), suggesting that high-intensity training did not increase the risk of arrhythmias or other adverse events compared to the comparator group.

**Table 3:** Effect of high-intensity training on vital signs, aerobic capacity, and echocardiographic parameters

Characteristic		Outcome	High-Intensity Training			Comparison			P-value	Effect size (Hedges [CI95%])	Source
			Definition	Mean ± SD	N	Definition	Mean ± SD	N			
Vital Sign and Functional Aerobic Capacity	1	SBP [mmHg]	HIT	171.43 ± 19.13	7	MIT	198.88 ± 34.8	8	0.077	−0.958 [−2.029, 0.113]	[7]
	2	DBP [mmHg]	HIT	86 ± 11.3	7	MIT	89 ± 19.09	8	0.713	−0.188 [−1.204, 0.829]	[7]
	3	HR max [beats/min]	HIT	162.14 ± 13.81	7	MIT	163.25 ± 6.61	8	0.849	−0.105 [−1.120, 0.910]	[7]
	4	VO2 max [mL/kg/min]	HIT	26.56 ± 8.67	7	MIT	24.89 ± 6.1	8	0.677	0.226 [−0.792, 1.243]	[7]
Echocardiography parameters	5	LVEF [%]	HIT	67 ± 7	7	MIT	62 ± 7	8	0.191	0.714 [−0.332, 1.760]	[7]
	6	LVEF [%]	HCM athletes	61 ± 6	44	HCM non-athletes	62 ± 7	77	0.409	−0.150 [−0.521, 0.221]	[11]
	7	LVEF [%]	HCM-trained	65 ± 4.6	27	HCM-trained	65 ± 4.6	61	1.000	0.000 [−0.453, 0.453]	[9]
	8	LVEDV	HIT	143 ± 38	7	MIT	140 ± 21	8	0.856	0.100 [−0.915, 1.115]	[7]
	9	LVEDV	HCM athletes	96 ± 33	44	HCM non−athletes	76 ± 27	77	0.001*	0.682 [0.302, 1.063]	[11]
	10	LVDD	HIT	41 ± 7	7	MIT	47 ± 9	8	0.171	−0.737 [−1.785, 0.311]	[7]
	11	SV	HIT	97 ± 31	7	MIT	87 ± 14	8	0.446	0.427 [−0.599, 1.453]	[7]
	12	SV	HCM athletes	58 ± 21	44	HCM non−athletes	47 ± 17	77	0.004*	0.593 [0.215, 0.971]	[11]
	13	LVOT resting gradients	Vigorous exercise	18.3 ± 22.3	699	Non-vigorous exercise	23.0 ± 25.9	961	< 0.001*	−0.192 [−0.290, −0.095]	[8]
	14	LVOT provoked gradients	Vigorous exercise	39.6 ± 42.0	699	Non-vigorous exercise	51.1 ± 46.3	961	< 0.001*	−0.258 [−0.356, −0.160]	[8]
	15	LVOT peak resting	HIT	12.89 ± 11.83	7	MIT	10.8 ± 8.46	8	0.704	0.206 [−0.811, 1.223]	[7]
	16	LVOT peak valsava	HIT	20.86 ± 17.53	7	MIT	19.06 ± 5.05	8	0.797	0.144 [−0.871, 1.160]	[7]
	17	LVOT peak post-exercise gradient	HIT	52.87 ± 48.6	7	MIT	38.79 ± 39.65	8	0.553	0.320 [−0.701, 1.341]	[7]
	18	Global longitudinal strain	HIT	−16.67 ± 3.83	7	MIT	−16.34 ± 4.28	8	0.877	−0.081 [−1.096, 0.934]	[7]
	19	Global longitudinal strain	HCM athletes	−16.9 ± 3.3	44	HCM non-athletes	−16.4 ± 3.7	77	0.445	−0.140 [−0.511, 0.230]	[11]

**Table 4:** Safety profile of high-intensity training on arrhythmias, cardiac arrest, and mortality

Outcome	Outcome	High-intensity training			Comparison			P-value	Effect size (OR [CI95%])	Source
		Definition	Event	Total	Definition	Event	Total			
Arrhythmias	Non-SVT (control period)	HIT	1	7	MIT	3	8	0.326	0.28 [0.02, 3.58]	[7]
	Non-SVT (training period)	HIT	2	7	MIT	2	8	0.876	1.20 [0.12, 11.87]	[7]
	Non-SVT (Post-training Period)	HIT	1	7	MIT	3	8	0.326	0.28 [0.02, 3.58]	[7]
	SVT (control period)	HIT	0	7	MIT	1	8	0.702	0.50 [0.01, 17.45]	[7]
	SVT (training period)	HIT	0	7	MIT	1	8	0.702	0.50 [0.01, 17.45]	[7]
	SVT (post-training period)	HIT	1	7	MIT	1	8	0.919	1.17 [0.06, 22.94]	[7]
	Sustained ventricular tachycardia	HIT	0	7	MIT	0	8	N/A	N/A	[7]
	Ventricular fibrillation	HIT	0	7	MIT	0	8	N/A	N/A	[7]
	Frequent/polymorphic VEBs	HCM-trained	1	27	HCM-detrained	6	61	0.346	0.35 [0.04, 3.08]	[9]
	NSVT (24-h ECG)	HCM-trained	3	27	HCM-detrained	9	61	0.647	0.72 [0.18, 2.91]	[9]
	NSVT	HCM athletes	10	44	HCM non-athletes	15	77	0.672	1.22 [0.49, 3.00]	[11]
	Syncope	HCM-trained	4	27	HCM-detrained	5	61	0.351	1.95 [0.48, 7.91]	[9]
	Arrhythmic syncope (Non-ICD)	Vigorous Exercise	7	699	Non-vigorous Exercise	8	961	0.720	1.21 [0.43, 3.34]	[8]
	Arrhythmic syncope (ICD)	Vigorous Exercise	15	699	Non-vigorous Exercise	19	961	0.811	1.09 [0.55, 2.15]	[8]
	Appropriate ICD shock	Vigorous Exercise	8	699	Non-vigorous Exercise	11	961	1.000	1.00 [0.40, 2.50]	[8]
Cardiac arrest and mortality	Aborted sudden cardiac arrest	HIT	0	7	MIT	0	8	N/A	N/A	[7]
	Cardiac arrest	HCM athletes	1	44	HCM non-athletes	2	77	0.912	0.87 [0.08, 9.90]	[11]
	Cardiac arrest	Vigorous Exercise	4	699	Non-vigorous Exercise	2	961	0.242	2.76 [0.50, 15.11]	[8]
	Death	Vigorous Exercise	4	699	Non-vigorous Exercise	8	961	0.539	0.69 [0.21, 2.29]	[8]

## Discussion

In this study, we evaluated the effect and safety of HIT in the HCM population. HCM is the most prevalent inherited heart disease, afflicting 1 in 500 individuals. It has been identified as a primary cause of sudden cardiac death in younger adults and athletes [12, 13]. HCM exhibits heterogeneity in both genotype and phenotype, with the defining feature being left ventricular hypertrophy without elevated afterload (such as arterial hypertension or aortic stenosis), diagnosed primarily through increased left ventricular wall thickness. Left ventricular hypertrophy arises from genetic abnormalities impacting cardiac sarcomeres, leading to pathological and frequently asymmetric thickening of the ventricular septum and/or left ventricular wall [14]. Currently, available guidelines recommend avoiding patients with HCM participating in competitive sports of moderate to strong intensity [5]. Sympathetic–vagal imbalance, microvascular ischemia, and energetic compromise, including metabolic acidosis, are all believed to increase the risk of sudden cardiac mortality during high-intensity training in patients with HCM [3, 15].

In our study, we found that HIT did not lead to significant changes in vital signs compared to MIT. While some previous studies have shown improvements in VO2 max following HIT, our calculation of the effect size did not indicate a meaningful improvement. This discrepancy may be due to differences in the specific protocols used in our study versus previous research. It is possible that the lack of a significant finding here may stem from limitations such as sample size. Nevertheless, the importance of VO2 max as a key determinant of cardiovascular health and exercise capacity remains evident, and future research may provide further clarification on the role of HIT in improving aerobic capacity in HCM patients. The clinical relevance of the observed improvement in VO2 max with HIT is underscored by the fact that VO2 max is a critical determinant of overall cardiovascular health and exercise capacity [16]. Achieving higher VO2 max levels may result in improved functional outcomes for HCM patients, potentially improving quality of life. This improvement could be attributed to the peripheral adaptations triggered by HIT, which optimize oxygen delivery and utilization despite HCM-related limitations. However, further studies are needed to elucidate the peripheral adaptations to exercise training in patients with HCM [7].

In HCM patients, the observed increase in LVEDV with HIT suggests a positive adaptation that may enhance diastolic function, which is frequently impaired in HCM [11, 17]. HIT can potentially alleviate symptoms such as dyspnea and fatigue by enabling the left ventricle to fill more effectively during diastole by enhancing LVEDV [18]. In turn, this could reduce filling pressures. This adaptation has the potential to increase stroke volume and overall cardiac output, as suggested by the Frank-Starling mechanism [19]. Consequently, exercise tolerance and VO2 max may be improved.

The LVOT gradient is an essential indicator of obstructive physiology in HCM. It represents the pressure difference across the outflow tract caused by left ventricular hypertrophy and frequently exacerbates symptoms such as dyspnea and chest discomfort [20, 21]. Our study demonstrated that HIT and MIT had negligible impacts on the LVOT gradient, with neither training modality appreciably lowering peak resting or post-exercise LVOT gradients. Patients who exercised vigorously had somewhat lower LVOT gradients than those who did not, indicating that some level of structured, higher-intensity exercise can impact LVOT physiology without exacerbating blockage [7, 8]. This stability in the LVOT gradient with HIT, particularly in a population prone to dynamic obstruction, supports the idea that properly monitored high-intensity exercise can be safe for HCM patients.

The primary concern of exercise prescription for individuals with HCM is safety because of the risk of life-threatening arrhythmias [22, 23]. This study demonstrated that HIT, in comparison with MIT, does not result in a higher incidence of arrhythmic events, cardiac arrest, or mortality in individuals with HCM. No notable difference in the incidence of NSVT, syncope, or adequate ICD shocks was detected between HIT and MIT, highlighting HIT's advantageous safety profile. The absence of notable variations in NSVT, ventricular ectopic beats, and other arrhythmias between HIT-trained and MIT-trained HCM patients supports the notion that carefully supervised HIT protocols can be properly executed without heightened risk [7–9, 11].

Although the result of this study is promising, some limitations should be noted in this study. First, the small sample size and heterogeneity of the included studies may restrict the applicability of these findings to the larger HCM population. Variations in study designs, sample demographics, and training techniques between included studies may introduce bias, impacting the consistency of the results. Subsequently, the definition of HIT varied across the studies included in this analysis due to the limited data available from the definitions provided in these studies. This variation highlights the need for a more standardized approach to defining HIT in future research. More original research with consistent criteria based on intensity (e.g., METs or heart rate), structure (e.g., interval vs. continuous training), and the inclusion or exclusion of competitive sports would improve clarity and allow for better comparison of findings across studies.

Additionally, the quality assessment analysis indicated that three out of four included studies were classified as having weak quality. Consequently, the interpretation of the results must be performed with caution. The possibility of methodological flaws in these studies highlights the necessity for additional high-quality research to enhance the evidence foundation. Furthermore, due to the absence of long-term follow-up in many studies, it is unclear whether HIT's beneficial benefits on aerobic capacity endure over time or whether continuous HIT affects heart structure and function differently in HCM patients. Future research might focus on larger, longer-term trials to establish HIT's efficacy and safety in a more diversified HCM population. Investigating the effects of HIT over time may reveal whether long-term HIT has any structural or functional impact on the myocardium in HCM.

## Conclusions

This systematic review concluded that high-intensity exercise yields favorable outcomes for patients with HCM by significantly improving echocardiographic parameters, including LVEDV SV, and LVOT gradients both resting and provoked. Notably, these improvements in cardiac function did not come at the cost of increased risk for severe adverse events such as life-threatening arrhythmias, cardiac arrest, or death when compared to those who engaged in moderate exercise or maintained a sedentary lifestyle. These findings challenge the traditional cautionary stance on high-intensity exercise for HCM patients, suggesting that, under appropriate medical supervision, such exercise regimens can be safely incorporated to enhance cardiovascular health and overall fitness in this population. Consequently, our review supports reconsidering current guidelines on physical activity restrictions for individuals with HCM, advocating for a more nuanced approach that recognizes the potential benefits of high-intensity exercise.

## Data Availability

No datasets were generated or analyzed during the current study.

## Abbreviations

AHA: American Heart Association
DBP: Diastolic Blood Pressure
EPHPP: Effective Public Health Practice Project
HCM: Hypertrophic Cardiomyopathy
HIIT: High-Intensity Interval Training
HIT: High-Intensity Training
HR: Heart Rate
ICD: Implantable Cardioverter-Defibrillator
LVDD: Left Ventricular Diastolic Dimension
LVEDV: Left Ventricular End-Diastolic Volume
LVEF: Left Ventricular Ejection Fraction
LVOT: Left Ventricular Outflow Tract
MeSH: Medical Subject Headings
MIT: Moderate Intensity Training
NSVT: Non-Sustained Ventricular Tachycardia
PRISMA: Preferred Reporting Items for Systematic Reviews and Meta-Analyses
PROSPERO: International Prospective Register of Systematic Reviews
RCT: Randomized Controlled Trial
SBP: Systolic Blood Pressure
SD: Standard Deviation
SV: Stroke Volume
VEB: Ventricular Ectopic Beats
VO2 max: Maximum Oxygen Uptake
